# Ethical and Social Values for Paediatric Health Technology Assessment and Drug Policy

**DOI:** 10.34172/ijhpm.2020.144

**Published:** 2020-08-11

**Authors:** Avram E. Denburg, Mita Giacomini, Wendy Ungar, Julia Abelson

**Affiliations:** ^1^Division of Haematology/Oncology, Department of Paediatrics, The Hospital for Sick Children, Toronto, ON, Canada.; ^2^Child Health Evaluative Sciences, Peter Gilgan Centre for Research and Learning, The Hospital for Sick Children, Toronto, ON, Canada.; ^3^Institute of Health Policy, Management and Evaluation, University of Toronto, Toronto, ON, Canada.; ^4^Department of Health Research Methods, Evidence and Impact, Centre for Health Economics and Policy Analysis, McMaster University, Hamilton, ON, Canada.

**Keywords:** Canada, Children, Health Technology Assessment, Public Values, Priority Setting, Drug Coverage

## Abstract

**Background:** Public policy approaches to funding paediatric medicines in advanced health systems remain understudied. In particular, the ethical and social values dimensions of health technology assessment (HTA) and drug coverage decisions for children have received almost no attention in research or policy.

**Methods:** To elicit and understand the social values that influence decision-making for public funding of paediatric drugs, we undertook a series of in-depth, semi-structured interviews with a stratified purposive sample (n = 22) of stakeholders involved with or affected by drug funding decisions for children at the provincial (Ontario) and national levels in Canada. Constructivist grounded theory methodology guided data collection and thematic analysis.

**Results:** Our study provides empirical evidence about the unique ethical and social values dimensions of HTA for children, and describes a novel social values typology for paediatric drug policy decision-making. Three principal categories of values emerged from stakeholder reflections on HTA and drug policy-making for children: procedural values, structural values, and sociocultural values. Key findings include the importance of attention to the procedural legitimacy of HTA for children, with emphasis on the inclusion of child health voices in processes of technology appraisal and policy uptake; a role for HTA institutions to consider the equity impacts of technologies, both in setting review priorities and in assessing the value of technologies for public coverage; and the potential benefits of a distinct national framework to guide drug policy for children.

**Conclusion:** Current approaches to HTA are not well designed for the realities of child health and illness, nor the societal priorities regarding children that our study identified. This research generates new knowledge to inform decision-making on paediatric drugs by HTA institutions and government payers in Canada and other publicly-funded health systems, through insights into the relevant social values for child drug funding decisions from varied stakeholder groups.

## Background

**Key Messages**

** Implications for policy makers**

- Current approaches to health technology assessment (HTA) and drug coverage decisions in Canada and comparable health systems are not well designed for the assessment of many child health technologies.
- Enhanced inclusion of paediatric expertise, as well as child and youth perspectives, in HTA processes could facilitate better incorporation of child health realities in drug policy decisions.
- Better incorporation of a range of identified social values in HTA and drug coverage decision-making, through deliberative public engagement and allied means, could strengthen the ethical basis of drug policy-making for children.
- Policy-makers should consider the development of a distinct national framework to guide HTA and drug policy for children in Canada; comparable research to elicit societal perspectives on HTA and drug policy for children is warranted in other publicly-funded health systems.

** Implications for the public**

Decisions about which drugs and health technologies to fund for children in Canada do not take sufficient account of social values from members of the public. This limits public input into whether and how to prioritize scarce health system resources for child health needs. Opportunities exist to better align drug coverage decision-making with values that society holds with respect to children. Our research generates an evidence-based framework of social values to help guide the development and implementation of drug funding policies for children. Use of this framework by health technology assessment bodies and policy-makers would support enhanced incorporation of a range of societal perspectives in the appraisal of paediatric drugs and therapeutics and thereby amplify public voice in drug coverage decisions affecting children. Future efforts to incorporate child and youth perspectives will be crucial additions to the evidence base for paediatric drug policies.

**Figure 1:**
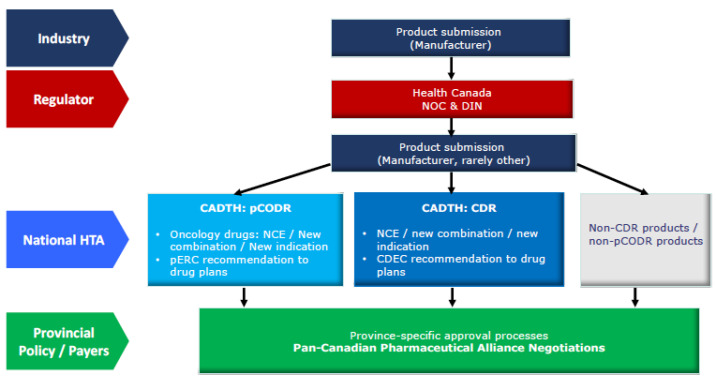
Drug Approval and Funding Process in Canada (Except Quebec). Abbreviations: CADTH, Canadian Agency for Drugs and Technologies in Health; CDEC, Canadian Drug Expert Committee; CDR, common drug review; DIN, drug identification number; HTA, health technology assessment; NCE, new chemical entity; NOC, notice of compliance — approval of drug safety and efficacy; pCODR, pan-Canadian Oncology Drug Review; pERC, pCODR Expert Review Committee.

Public policies on the regulation and funding of medicines for children vary considerably across developed health systems.^1-3^ Health technology assessment (HTA) institutions and processes are now central to public drug funding decisions in a growing array of developed health systems, including Canada.^4^ Technology assessments by the Canadian Agency for Drugs and Technologies in Health (CADTH) have become an influential determinant of the inclusion of novel drugs and therapeutics on provincial drug formularies.^5^ In the context of mounting cross-provincial engagement on drug pricing negotiation and coverage decisions through the Pan-Canadian Pharmaceutical Alliance, CADTH’s HTA recommendations play an increasingly important role in guiding cross-provincial policy harmonization (Figure 1).^6^

However, current approaches to HTA and drug policy-making in most countries take little account of the unique features of child health and illness.^7,8^ In particular, the ethical and social values dimensions of HTA and drug coverage decisions for children have received almost no attention in research or policy, despite their stated importance as a foundational component of HTA in most jurisdictions with public drug funding programs.^9,10^ This article explores the social values that influence decision-making for public funding of paediatric drugs, through analysis of interviews with stakeholders involved in or impacted by HTA and policy-making for child health technologies at the provincial (Ontario) and national levels in Canada. It contributes novel data to inform the design of prioritization and assessment frameworks for paediatric drugs and health technologies, with direct policy relevance to healthcare priority-setting bodies and government funders in Canada; it may also have implications for comparable health systems internationally.

## Methods

###  Data Collection

 Our study employed a constructivist grounded theory methodology. We conducted in-depth, semi-structured interviews with a stratified purposive sample (n = 22) of stakeholders involved with or affected by drug funding decisions for children from January-April 2018. The sample was comprised of a broad range of roles relative to HTA processes in Canada, including: parents of children with cancer and other chronic diseases from a range of Canadian provinces (PAR; n = 4); health professionals (physicians, allied health, pharmacists, bioethicists) involved in the care of such patients in Ontario (HEA; n = 7); professionals at national regulatory and HTA institutions in Canada (PRO; n = 4); and provincial policy-makers involved with drug coverage decisions in Ontario (POL; n = 7). Non-English speakers were ineligible to participate. We identified potential participants through grey literature review, institutional scans of relevant hospitals, HTA organizations and government websites, and referral from other stakeholders. We obtained written informed consent from participants prior to the conduct of interviews. The interview guide was developed based on prior literature review [A. E. Denburg, M. Giacomini, W. Ungar, J. Abelson, unpublished data, 2020],input from study team members, and iterative refinement based on emergent interview data; we developed distinct versions of the guide for parents, health professionals, and regulators/policy-makers. Interviews were audiotaped, transcribed verbatim and inductively coded using NVivo 11 software (QSR International, Ltd.). Data were anonymized and de-identified to protect participant confidentiality.

###  Data Analysis

We undertook iterative sequential phases of data coding, moving from open through theoretical codes, with constant comparative methods employed to refine codes, establish analytic distinctions, and capture emergent themes.^11^ Theoretical saturation was pursued through ongoing conduct of interviews to pursue salient themes as they emerged, which informed the development of conceptual and practical insights on social values related to child HTA. We sought saturation both within and across strata, as many of the concepts and themes explored retained relevance across participant roles and perspectives.

Theory on ‘technology-as-policy’ and the sociopolitics of health technologies served as sensitizing concepts for our inductive coding and data analysis.^12-14^ The notion of technology-as-policy invokes the inherently political nature of all technologies: “Technologies not only get things done, like policies, they also change what gets done, how and by whom it gets done, and who gains or loses as a consequence.”^15^ It underscores the power dynamics created and mediated by discrete technologies, and the moral implications of their development, use and disuse. Building on this conception of technology, we leveraged a theoretical framework on the sociopolitics of health technologies, which emphasizes assessment across four fundamental domains – actors, resources, knowledge, and power – to inform our understanding of the concepts and themes that emerged from our data.^14^ Sensitizing concepts in grounded theory are interpretive devices and provide a starting point for analysis, rather than a prescriptive schema for thematic interpretation and theory development. We employed the domains in this pre-existing framework as background concepts against which our emerging data were interrogated.^16^

## Results

Three main types of values emerged from stakeholder reflections on HTA and drug policy-making for children: *procedural* values, *structural* values, and *sociocultural* values.

###  Procedural Values

We define procedural values as those that relate to the processes underlying HTA and health system priority-setting on drugs for children. Participant concerns with the normative dimensions of *how* such systems of decision-making operate stood out. Legitimate ends were repeatedly premised on legitimate means: “*I think if the process is perceived as fair, people have less of a problem with the decision than if the process is perceived as unfair*” (PRO2). Procedural fairness was a recurrent theme:

“*I think you have to try and develop a fair process … Good solid base for decision-making, a fair process, a transparent process – and then make the best decision you have at the time with that information and be open to amending that if new information comes along*” (POL4).

Interestingly, children’s right to participate in the process of valuing health technologies was among the most prominent values endorsed. Participants questioned the routine lack of child voices in health and social policy decision-making about children, and proposed a collective challenge to current HTA paradigms to do better on this front: “*Patient preferences [are] very child-relevant because God knows what that means, when they don‘t have a voice*” (HEA2). The inherent limitations associated with preference elicitation from proxy decision-makers, including parents, were emphasized:

“*When you start moving into paediatrics, and you’re using substitute decision-makers, and other caregivers, I think the quality of some of that information can diminish, and just introduces a number of other challenges into the process*” (PRO1).

 A number of participants gave voice to the potential for unique and distinctly valuable insights from enhanced child participation in HTA, citing research on the elicitation of policy priorities among young children:

“*What [researchers] heard [from children] was so different from what they expected, and so showed them that the way they would have prioritized where they put the money would have been so wrong if their goal was really meeting the needs of these little ones*” (HEA7).

 Others, by contrast, questioned the wisdom and feasibility of incorporating child and youth voices into HTA and drug policy-making. One participant referenced emerging science on continued neurodevelopment beyond adolescence, and its implications for executive function and corollary conceptions of capacity even in this age group:

“*[Consider] this newer literature on the young person’s brain developing into their 20s and risk assessment being one of the last things to develop. So, you know, ‘if I can’t play soccer…then life is over*’” (HEA5).

 Nevertheless, most participants favoured enhanced efforts at child participation in the assessments and decisions that govern their access to health technologies.

 A related theme about legitimate perspectives to incorporate into HTA processes centred on the tension between access to specific drugs and responsible societal resource stewardship. A number of participants identified competing interests at the patient and community levels in respect of coverage decisions:

“*It’s hard enough to say no in a circumstance when, you know, the data are poor and you really can’t justify recommending something. But it’s an even harder thing to say, you know what, this just doesn’t work with our values because this is where that really pointy part of individual rights really bumps up against society’s rights or society’s interests*” (POL6).

Some argued for the primacy of collective societal values above patient and professional ones in the realm of public funding priorities – “*I don’t think it should be the values of folks around the table making the decision – it’s not my personal values that matter, it’s the values of society*” (POL3) – and argued for enhanced public input into decision-making processes.

###  Structural Values

The existence and impact of *structural* values – those internal to and formative of HTA decision-making frameworks, broadly inclusive of clinical evidence appraisal, pharmacoeconomic evaluation, and consideration of patient values – also emerged as a coherent theme in the data. Participant reflections on life-course potential and fair innings, equity and unmet need, and the moral calculus of economic arguments were all central to interpolations of standard HTA logic for child health. The construct of the family also emerged as a structural value in specific instantiations, notably in relation to economic methods and the enhanced incorporation of social context into HTA.

####  Life Years Gained: Potential and Fair Innings

 Life-course perspectives were frequently invoked to justify approaches to HTA for child health technologies that diverged from, or directly complicated, established HTA paradigms. The idea of ‘life years gained’ stood out in this context. Value propositions for paediatric drugs and health technologies were frequently framed and scaled in line with their capacity to yield gains in future years of life:

“*I do think there are different ways to look at the technology when it’s used in paediatric healthcare. In some ways I think of it as more akin to preventive medicine. Because presumably if you treat people well and can extend their lifespan they have a lot more life to gain*” (PRO1).

 Participants often qualified their view about life years gained with ideas about potential and fairness. The most common formulations of potential were couched in terms of collective economic gains, be they to the health system or to society at large:

“*The economic benefits of productivity gains to be had with good child health – that on its own should be a reason why governments should care. Because if your kids don’t do well then they can’t be productive citizens and they can’t contribute to your GDP [gross domestic product]*” (POL4).

 However, participants also made powerful allusion to the personal, familial and communal benefits reaped from childhood potential realized, or alternately, squandered:

“*[My son] was 3 when he died. He didn’t get to play soccer, he didn’t get to go to high school, he didn’t get to go to university. He doesn’t get to do all the things his twin brother is doing”* (PAR1).

 Related to ideas about potential were notions about fairness. A wide range of participants referenced the existential value of experience across the arc of a life, and the injustice of a child deprived of such experience.

“*[When my mother died] it was sad, it was too early, there was a lot of things she still had to give but she had a full life. There’s a different moral imperative in terms of [children]. I wish they were both still here but neither of them are – but there’s a different moral imperative around a 3-year old dying and 80-year old dying and what they got to do*” (PAR1).

####  Unmet Need

 The trope of unmet need was also leveraged in equity-based arguments for tailoring HTA to child realities and needs. Participants spoke to the relative lack of treatment options for children – as a result of gaps in clinical evidence, drug development, or licensed indications – and highlighted the equity implications of this status as ‘therapeutic orphans.’ This unmet need was framed by some as an intrinsic justification for the prioritization of health technologies for children – not to supplant other means of assessing value, but to complement them:

“*On the pediatric side, just given the fact that many of the current therapies often don’t have a pediatric indication…there may be an unmet need. [We must] identify the gaps in the current treatments, from a number of different factors, that make it important for us to bring forward a positive funding recommendation for this drug*” (PRO4).

 An array of stakeholders noted that the concept of unmet need is presently incorporated as a component of certain HTA frameworks, but implied that its form and reach remain hazy:

“*Things might have similar budget-impact, similar cost-effectiveness, similar, you know, marginal extension of life. But then there are the sort of gut things about, well, so all of that may be true, but it’s more important to have an option to give someone than to pile something else on existing options, and how do you quantify that*?” (POL6).

A few participants connected unmet need to the idea of hope, suggesting that, particularly for severe or life-threatening conditions, the availability of treatment options *per se* had inherent value. Some noted the value of individual therapies in their role as one in a sequence of options, insofar as they sustain such hope in the context of rapidly evolving scientific knowledge:

“*Sometimes [the therapy] will be a bridge – you’re just trying to get somebody to the point where they’ll be the candidate for some other [treatment]*” (HEA2).

 One participant noted the potential for unanticipated benefits from novel drugs in diseases and populations distinct from their initial indication, and framed such spillover as fuel for the forward march of medical knowledge:

“*We’ve known for centuries that discoveries in one group of patients will very often bring themselves back to another group of patients that was really quite unanticipated. So if we close some of those doors, I fear that we actually slow down the progress of our ability to support humanity generally*” (HEA1).

####  Family

 Finally, the construct of the family was a unique theme that emerged from participant reflections on the normative structure of HTA for children. Family context and impact were deemed essential components of the value propositions attached to the assessment of childhood drugs and technologies. The idea of the family was frequently invoked in relation to the economic methods that underpin value assessments of child health technologies. Participants spoke to the decreased societal and economic productivity of family members of children with severe or chronic illness, and the lack of capture of these dynamics in standard economic assessments:

“*When a child dies, the families that I see, that are my friends who have lost their son or daughter, their ability to function in society and the world just becomes so immensely impaired*” (PAR3).

 They also repeatedly noted the importance of incorporating family context and perspectives in measures of utility among children:

“*An unhealthy child is generally an unhealthy mother and, not uncommonly, an unhealthy father and siblings as well. So, the notion of unit of analysis, I think, is very germane to childhood*” (HEA6).

 A few participants noted the potential to incorporate the little-heard voices of bereaved parents in child HTA. They referenced studies that elicit perspectives from parents following the death of their children, the perspectival changes alluded to by these parents, and the value in juxtaposing such views to those of patients and families currently engaged in efforts to access therapies:

“*Bereaved family members within six months of the death of the patient…had such a different perspective, having gone through to the end, and then looking back...*” (HEA7).

 Participants also affirmed the value of parental perspectives in assembling grounded, real-world knowledge of child health technologies. The quotidian impacts of a given therapy – on the child, on the surrounding family – were felt to be poorly captured or prioritized in current HTA frameworks:

“*It also has an impact on the family unit, just with trying to manage diets, and that’s not just for the one kid in the house, it’s for the entire family. So it goes beyond just the individual and could impact, you know, parents’ quality of life, ability to retain jobs, all that sort of stuff*” (PRO1).

###  Sociocultural Values

We identified a final strain of values that spoke to how broader social and cultural values relate to, shape, and condition responses to drug policy decision-making for children. These *sociocultural* values assembled into three main themes: culture, equity, and distinction.

####  Distinction

The notion that children are distinct, or unique, in sociocultural terms coloured many participants’ reflections on paediatric drug policy and access. Expressed in varied ways, the sentiment that children constitute a separate and special social group, and that a number of the normative considerations in child health policy are therefore *sui generis*, was widely held and forcefully stated by participants. As a consequence of this perceived distinction, a range of participants identified a moral imperative for society to protect and promote the health and well-being of children:

“*Societies are judged by how they treat the elderly, the infirm, and the children. When the infirm are also the children, I think there is a double ethical responsibility by society*” (PAR2).

 For some, this sense of duty derived from a parental impulse to nurture, transposed from the individual to the collective. For others, it was connected to an inchoate conviction in the fair innings argument described above. For others still, this societal imperative attached to our deep, instinctual drive for species survival in evolutionary terms. Some saw this instinct as fundamentally human, and the corollary imperative as something shared across diverse human societies:

“*[If] society believes there to be different values in child health, we should simply say that. I don’t think there’s any shame or anything to be upset about. I think it may be a societal preference that we have, that many societies in fact have. If you think about it from even a pure survival-of-the-species perspective, if we’re not caring for our young, our species is toast*” (POL3).

The effect of normatively distinguishing children was, for some, grounds to justify paediatric exceptionalism in policy. This moral obligation – called by one participant the “*founding principle of a compassionate society*” (HEA1) – was directly tied to drug access by a number of stakeholders, including societal willingness to pay for children’s health technologies:

“*There needs to be a higher [cost] threshold when dealing with children than with adults. And I don’t think you’d get a strong argument from anybody against that*” (HEA4).

####  Equity

Entwined with their recognition of childhood distinction, many participants identified the paradox of children’s relative marginalization in society. This perceived marginalization – encapsulated in the trope of children as ‘*an invisible minority’* (HEA2) – was predicated on ideas about childhood vulnerability. Some framed this vulnerability as a lack of capacity for self-advocacy, and identified a corollary societal obligation to protect the interests of children in legal and policy fora:

“*The vulnerability of not being able to advocate for yourself, calls on a different level, perhaps, for stewardship that takes into account that you don’t play equally because this group can’t be advocating in the same way*” (HEA4).

 Relative inequity in drug access between children and adults was traced to this intrinsically powerless state, a consequence of both biological and political vulnerability:

“*Equity is an even more important consideration in children than adults because they have very little say in their place in society, their socioeconomic position*” (POL5).

 Anticipating comparable claims to political privilege from other vulnerable or marginalized groups, a number of participants sought to stress categorical distinctions of childhood vulnerability. Notions of innocence coloured participants’ portraits of inequity in drug access and health outcomes for children, and their attempts to distinguish it. For some, children’s near-total lack of responsibility for their health state justified their prioritization in funding decisions. Others located a key source of difference in children’s unique and evolving developmental state:

“*Sometimes the pushback is yeah, so, you know, do we make [drug policy] different for women, [ethnic] minorities, immigrants? No. But there is a discrete developmental population in which devastating diseases occur. They occur rarely but they are critical in child health*” (HEA4).

####  Culture

While shared convictions about both children’s special status and relative marginalization seemed to pervade many of the interviews, there was also a sense that culture could modulate collective mores about children in a given society. A few stakeholders noted the bounded sociocultural lens through which they view children in relation to society. They intimated that dominant narratives about children in our society might appear parochial when viewed in a global context, and further, that such narratives might not adequately represent the nuance and variegation of cultural perspectives even *within* our society, or others like it:

“*[Take] a cultural group that doesn’t have that same lens…[and] puts priority on the elderly over kids…that culture gives a lot of deference to the contributions seniors have already made through their lifetime, and kids – ‘what have they done that’s worthy of the extra point*?’” (HEA4).

 In addition to identifying varied ideas about children’s vulnerability and need for protection in different cultural contexts, a few stakeholders emphasized the role of culture in notions of fairness. They challenged the uniformity of cultural attachments to the ‘fair innings’ argument:

“*This fair innings notion seems to be a very - I don’t know if it’s fair to say Western, but may be North American-centric type of framework and that whereas, you know, it might seem sort of obvious place to start to some, we can’t make that sort of presumption definitely as we’re much more of a global multicultural society*” (HEA6).

One participant drew a line from the divergence of cultural norms on age-based prioritization to legal provisions protecting against age-based discrimination. Specifically, the role of the *Canadian Charter of Rights and Freedoms *was invoked to describe the potential limits of preferential funding for paediatric drugs, and, perhaps more importantly, to underscore the competing moral principles that might reasonably attach to governmental policy on drug funding. As against this, some referenced provisions around minimal impairment in *Charter* rights denial: the need to prove that the “*harm of denying that right is less bad than the harm that’s going to come from the activity*” (HEA6) – namely, prioritizing children in drug resource allocation. Others pointed to inherent states of vulnerability and marginalization in childhood as counterweights to claims of age-based discrimination in western liberal democratic legal philosophies:

“*If you can make the case that you’re part of a group that has been traditionally marginalized or treated sort of with less benefit, then to try and compensate or to even push higher than equality would be defensible without falling short and being accused of discrimination on basis of age*” (HEA7).

## Discussion

###  Policy and Practice Implications

**Figure 2:**
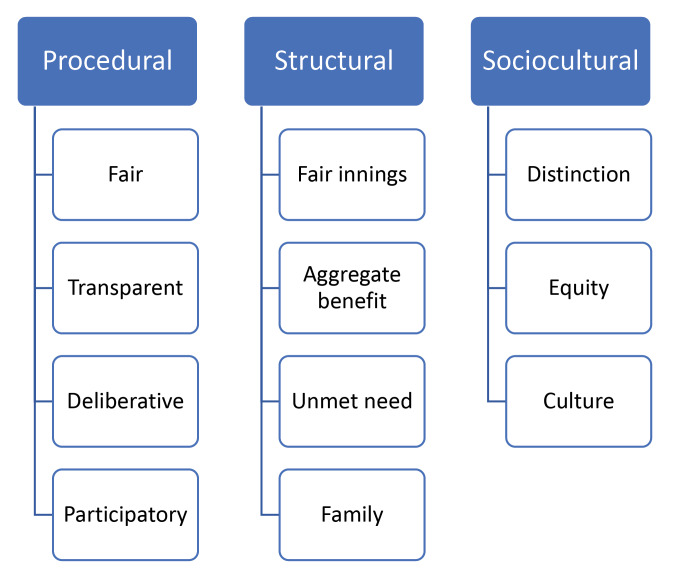
Values Typology for Health Technology Assessment and Drug Coverage for Children.

Broad endorsement of the need for an HTA paradigm tailored to children – one that takes account of both their intrinsic differences and their distinct place in society – emerged as a signal theme from our interviews. This theme issued from, and was justified through, participant reflection on the social values that underpin drug policy processes and decisions for children. Our study generates insights into the social values relevant to such decisions from a range of key stakeholder groups, and assembles this evidence into a typology of values that can be used to assess HTA paradigms and drug funding decisions for children in a range of health system and societal contexts. Three categories of values emerge from the data: procedural values, structural values, and sociocultural values (Figure 2). Applying this values typology to phases of the HTA process and elements of health system context enables a rich normative analysis of key concepts related to drug funding for children.

####  Procedural Values

Procedural values relate to the processes underlying HTA and health system priority-setting on drugs for children. Given the thorny and intensely contested moral choices involved in HTA and drug coverage decisions for children, participants often hewed to procedural values to ground their arguments on ethical priority-setting. Assertions of procedural legitimacy were tied to a cluster of related ideas: participation, deliberation, transparency, collective values, and the push-pull between orthodox methods and moral instincts. The moral relevance of process to sequential phases of technology prioritization, assessment, recommendation and implementation was asserted time and again by participants. In large part, this relevance related to voice. The formal inclusion of child health scientists, practitioners, patients and, notably, broader publics into HTA priority-setting and evaluation was seen as a corrective to industry-dominated submission processes and assessment frameworks built for adult disease realities, respectively, both of which create intrinsic bias against the system uptake of health technologies for children.^17,18^

Children’s right to participate in the process of valuing health technologies is a novel and challenging theme in this domain.^19^ A number of participants noted the strong normative and jurisprudential foundations for child participation in the United Nations Convention on the Rights of the Child, which defends children’s right to participate in decision-making processes affecting them.^20^ At the same time, many recognized the challenges to doing so in the HTA space. The tension between capacity and participatory rights was, for many, not easily resolved. Moreover, the practical means for enabling this participation and incorporating its results were not always clear to participants. When and how to assimilate child values and preferences into the patient input solicited for HTA, or into the methods used to assign health state utilities in the development of pharmacoeconomic models to inform it, were questions posed but not answered.

 A related theme to surface was the tension between the incorporation of individual and societal perspectives in drug policy-making for children. The value of personal experience – of disease, of treatment, of specific social context – was deemed by many an integral part of HTA reviews. Running parallel to this was a recognition by some stakeholders of the often-indissoluble conflict between individual and community priorities. Many felt that the arithmetic of societal values on drug coverage would differ for children, and that public voice should play a greater role in value assignment and priority-setting, in light of intrinsic evidentiary limitations attached to child HTA.

####  Structural Values

Structural values are those formative of the HTA framework itself. In this domain, participants emphasized a number of values of outsized importance to HTA for child health technologies. The simple fact of youth, of years of life left to live, animated most participants’ moral reasoning on HTA for children. Interestingly, the issue of disability did not often arise: participants gave little consideration to the value of life with disability, nor assumptions about the same incorporated into standard methods for health economic evaluation. Layered on the arithmetic of life years were two principles that refined the moral calculus for many stakeholders: potential and fairness. Recognition of a child’s latent potential, in social and economic terms alike, was central to participants’ views on the need to do HTA differently for children. This conviction was expressed in relation to both personal and public spheres of life. Many participants connected ideas about potential to notions of fairness. The intersection of these values is captured in the philosophical concept of ‘fair innings’: it holds that everyone should have the chance to live the whole of a life, and that we should therefore give priority to those who have had less chance to do so. In the language of health economics, the ‘fair innings’ proposition posits that the less quality-adjusted life years one enjoys from birth till death, the worse off one is.^21^ Many participants displayed intuitive affiliation with the idea of ‘fair innings’ and leveraged it as a justification for calibrating HTA to weight years gained early in life more heavily than those gained later.

 Participants’ additional emphasis on unmet need as a fundamental reality for many childhood diseases underscores the lack of formal attempts in HTA institutions or literature to take account of the systemic issues that condition therapeutic need in child health – including limited data on clinical effectiveness and a paucity of licensed indications – and to incorporate these dynamics in drug reviews for paediatric indications. The perceived combination of historical exclusion and gathering momentum in the paediatric drug space coloured many participants’ reflections on drug access for children, and placed unmet need alongside life-course potential, fair innings, and aggregate life years gained as core justifications for a child-specific HTA framework.

 Finally, ideas about family context and impact emerged as distinguishing concerns for many participants. Many stakeholders asserted that explicit incorporation of child and family perspectives had disproportionate importance in the assessment of paediatric drugs for public coverage. Things little considered in adult health emerged as crucial determinants of the impact and acceptability of a given therapy among children: formulation specifics (dosage form, site of administration, palatability) and side effect profiles (both short- and long-term) often acted as hinge points for the immediate and future quality of life of the child and family.

 Proposals to better incorporate these dimensions of child life and illness into HTA ranged from alternative methods for developing health state utilities to structured procedural incorporation of parent and public voices at various points throughout the HTA continuum, from priority setting to evidence appraisal. How precisely to achieve this formal incorporation of family dynamics into HTA paradigms is less apparent. But a sense that something is being missed in established methods for economic evaluation of child health technologies is clear. Stakeholders from across the range of perspectives represented asserted that HTA institutions need to spend time considering how to optimize economic assessments to take account of familial impacts. In short, ‘the family’ emerged as an insufficiently considered, but deeply important, mediator of the relationship between children and the health technologies they need, and a co-recipient of the benefits and burdens attached to them. There is evident need for HTA principles and processes better calibrated to the realities of children’s social context, with family at the centre.

The impact of structural values on technology prioritization and evaluation thus emerged as an important determinant of access to child health technologies. Practical realities governing the choice of technologies for assessment by HTA institutions, coupled with the power of particular sets of actors, shape the production of knowledge on, awareness about, and uptake of, competing health technologies.^22^ Again, industry interests and voice predominate, rendering financial calculus an outsized determinant of priority. Alternative values for technology selection – such as equity, need, disease severity, potential impact, and the presence of treatment alternatives – would help bring priority-setting by HTA institutions in line with societal values, including those that would favour the evaluation of health technologies for children.^23-26^ In terms of the parameters for technology evaluation per se, increased emphasis on considerations such as fair innings, unmet need, and family context could help rebalance the moral bases upon which HTA assessment rests.^21,27^ Explicit consideration of the distributional impacts of priority-setting and funding recommendations by national and provincial HTA institutions would go a way towards mitigating this imbalance. More routine use of equity as a frame for technology prioritization and evaluation is a viable first step.^28,29^

####  Sociocultural Values

 Sociocultural values capture how wider social and cultural values condition drug policy for children. Participant reflections on the role of culture in mediating public perceptions about the place of children in society, and the collective duties owed them, constituted an important challenge to easy assumptions about society’s allocative preferences. While not eschewing the biological and social differences ascribed to children across the interviews, they troubled easy assimilation of these distinctions into social policy – at least in the absence of careful incorporation of a range of societal perspectives. Rather than overturn claims of inequity or distinct need attached to children, this challenge adds valuable complexity to such claims, underscoring the need for robust processes and justificatory frameworks to ground allocative decisions on paediatric drugs.

 The discrepancy between conceptions of child distinction and priority, on the one hand, and constrained access to drugs, on the other, may evince a lack of intimate knowledge about child health realities, and the development of drug policies and systems predicated on that ignorance. The play of other overriding instincts that might blunt individual and social norms attached to children is also evident: the market dynamics of paediatric drug development and the realities of political voice in democratic institutions are two ready examples. Even so, our results affirm both a moral basis and practical opportunities for reform of paediatric drug systems and policies in line with societal beliefs about the unique status of children.

The legitimacy of paediatric exceptionalism, the importance of equity, and the space for cultural diversity in drug policy for children are key normative issues that demand more attention. Ideas about children’s disadvantage and unmet need in the health technology space imply the importance of equity as an organizing principle for paediatric drug funding decisions.^30^ Participant reflections in the sociocultural realm also reveal fundamental claims to childhood as an ontologically and thus morally distinct state, one invested with unique societal meaning. On the force of such claims – including allusions to inherent vulnerability and corollary societal duties to protect – a number of participants advocated for paediatric exceptionalism in drug policy. The intent of this exceptionalism seemed less an attempt to devalue alternative experiences of inequity, or related claims to priority, than a disavowal of zero-sum competition between them. The recurrent links made by participants between childhood vulnerability and inequity of access to drugs served, in aggregate, as grounds for the explicit incorporation of equity considerations in paediatric drug funding decisions. The practicalities of implementing such exceptionalism were left largely unexamined. Moreover, a few participants challenged these claims, noting the varied imprint of culture on perceptions about the value and place of children in society. Nevertheless, a strong narrative emerged around the need to think distinctly about children’s health technologies and access to them, and to consider ways of embedding such distinction in practice.

###  Strengths and Limitations

 Our study provides robust empirical evidence about the unique ethical and social values dimensions of HTA for children, and generates a novel social values typology for child HTA. We employ this typology and associated concepts to produce insights into how to understand and improve drug assessment and policy-making for children in public health systems, with particular emphasis on the Canadian context. Remarkably, almost no evidence exists on the principles that structure drug funding decisions for children in any health system context, including the social values that animate those decisions. This study serves as a first in-depth foray into the role of such values in HTA, with sufficient context-specificity to yield both foundational and particular knowledge for policy.

 Limitations in our study relate mainly to the perspectives reflected in our data. We did not formally sample members of the general public, nor undertake stratified sampling of other segments of the population with unique drug access experiences, such as those with rare diseases. We also did not interview children themselves. Involving these voices in future analyses of child HTA may yield novel insights. Our relatively small sample size, bounded stakeholder groups, and province-specific Canadian case study arguably limit applicability of the study’s findings to other jurisdictions, within Canada and beyond. In particular, the social values constructs that emerged from our sample are contingent on the wider values at play in Canadian society, and may not accurately reflect the range of sociocultural instincts or moral convictions in other societies, even those with similar economic, political and cultural histories. While variation doubtless exists, allied work by our team to systematically review and synthesize academic literature on the moral foundations of child health and social policy suggests broad consonance of values related to children and health across a wide range of societies [A. E. Denburg, M. Giacomini, W. Ungar, J. Abelson, unpublished data, 2020].Future work could focus on extending such social values and policy analyses to cross-country comparisons of HTA for children.

## Conclusion

 Current approaches to HTA are not well designed for the realities of child health and illness, nor societal priorities relative to children. Our study generates new knowledge to inform policy-making on paediatric drugs, relevant to both HTA institutions and government payers, through insights into the relevant social values for child drug funding decisions from varied stakeholder groups. The resultant typology of procedural, structural, and sociocultural values is applicable to the evaluation of prevailing HTA paradigms and drug funding decisions for children in a range of health system and societal contexts.

 Our use of this typology to catalogue and understand the play of social values across phases of the HTA process and the broader health system context yields a few key insights: (1) the importance of attention to the procedural legitimacy of HTA for children, with emphasis on the inclusion of child health voices in processes of technology appraisal and policy uptake; (2) a role for national and provincial HTA institutions to consider the equity impacts of technologies, both in setting review priorities and in assessing the value of technologies for public coverage; and (3) the potential benefits of a distinct national framework to guide drug policy for children. The insights generated have direct bearing on the Canadian health system, with particular applicability to Ontario, but also yield fundamental knowledge about the normative dimensions of HTA for children of value to drug policy-making in other publicly-funded health systems.

## Ethical issues

 The Hamilton Integrated Research Ethics Board affiliated with McMaster University approved this research.

## Competing interests

 Authors declare that they have no competing interests.

## Authors’ contributions

 AED led the design of the study, conduct of the interviews, coding and analysis of the qualitative data, and initial draft of the manuscript. JA, MG, and WU contributed key input on study design, the development of data collection tools and coding structures, and the interpretation of analytic results, and provided critical revisions to the manuscript.

## Funding

 During the period of this research, AED was supported by grants from the Canadian Institutes of Health Research, the Canadian Child Health Clinician Scientist Program, and the Pierre Elliott Trudeau Foundation.

## Authors’ affiliations

^1^Division of Haematology/Oncology, Department of Paediatrics, The Hospital for Sick Children, Toronto, ON, Canada. ^2^Child Health Evaluative Sciences, Peter Gilgan Centre for Research and Learning, The Hospital for Sick Children, Toronto, ON, Canada. ^3^Institute of Health Policy, Management and Evaluation, University of Toronto, Toronto, ON, Canada. ^4^Department of Health Research Methods, Evidence and Impact, Centre for Health Economics and Policy Analysis, McMaster University, Hamilton, ON, Canada.
